# Development and validation of a prediction model for coronary heart disease risk in depressed patients aged 20 years and older using machine learning algorithms

**DOI:** 10.3389/fcvm.2024.1504957

**Published:** 2025-01-09

**Authors:** Yicheng Wang, Chuan-Yang Wu, Hui-Xian Fu, Jian-Cheng Zhang

**Affiliations:** ^1^ Shengli Clinical Medical College of Fujian Medical University, Fujian Medical University, Fuzhou, Fujian, China; ^2^ Department of Cardiovascular Medicine, Fuzhou University Affiliated Provincial Hospital, Fuzhou, Fujian, China; ^3^ Department of Cardiology, Fujian Provincial Hospital, Fuzhou, Fujian, China; ^4^ Department of Cardiology, Youxi County General Hopital, Sanming, Fujian, China; ^5^ Department of Cardiology, Changji Prefecture People’s Hospital in Xinjiang Uygur Autonomous Region, Changji, Xinjiang, China

**Keywords:** depression, machine learning, prediction model, coronary heart disease, National Health and Nutrition Examination Survey (NHANES)

## Abstract

**Background:**

Depression is being increasingly acknowledged as an important risk factor contributing to coronary heart disease (CHD). Currently, there is no predictive model specifically designed to evaluate the risk of coronary heart disease among individuals with depression. We aim to develop a machine learning (ML) model that will analyze risk factors and forecast the probability of coronary heart disease in individuals suffering from depression.

**Methods:**

This research employed data from the National Health and Nutrition Examination Survey (NHANES) from 2007–2018, which included 2,085 individuals who had previously been diagnosed with depression. The population was randomly divided into a training set and a validation set, with an 8:2 ratio. Univariate and multivariate logistic regression analyses were employed to identify independent risk factors for coronary heart disease in individuals with depression. Eight machine learning algorithms were applied to the training set to construct the model, including logistic regression (LR), random forest (RF), gradient boosting machine (GBM), support vector machine (SVM), extreme gradient boosting (XGBoost), classification and regression tree (CART), k-nearest neighbors (KNN), and neural network (NNET). The validation set are used to evaluate the various performances of eight machine learning models. Several evaluation metrics were employed to assess and compare the performance of eight different machine learning models, aiming to identify the most effective algorithm for predicting coronary heart disease risk in individuals with depression. The evaluation metrics applied in this study included the area under the receiver operating characteristic (ROC) curve, calibration curve, Brier scores, decision curve analysis (DCA), and the precision-recall (PR) curve. And internally validated by the bootstrap method.

**Results:**

Univariate and multivariate logistic regression analyses identified age, chest pain status, history of myocardial infarction, serum triglyceride levels, and education level as independent predictors of coronary heart disease risk. Eight machine learning algorithms are applied to construct the models, among which the Random Forest model has the best performance, with an (Area Under Curve) AUC of 0.987 for the random forest model in the training set, and an AUC of 0.848 for the PR curve. In the validation set, the random forest model achieves an AUC of 0.996, and an AUC of 0.960 for the PR curve, which demonstrates an excellent discriminative ability. Calibration curves indicated high congruence between observed and predicted odds, with minimal Brier scores of 0.026 and 0.021 for the training, respectively, reinforcing the model's ability to discriminate. Set and validation set, respectively, reinforcing the model's predictive accuracy. DCA curves confirmed net benefits of the random forest model across. Furthermore, the AUC of the random forest model was 0.928 after internal validation by bootstrap method, indicating that its discriminative ability is good, and the model is useful for clinical assessment of the risk of coronary heart disease in depressed people.

**Conclusion:**

The random forest algorithm exhibited the best predictive performance, potentially aiding clinicians in assessing the risk probabilities of coronary heart disease within this population.

## Introduction

Depression is recognized as the most prevalent mental disorder worldwide, affecting millions of individuals across diverse demographics and cultures (1). Statistical data indicates that around 190,000 people in the United States are diagnosed with depression annually (2). This mental health condition manifests through various key physical symptoms, including fatigue, persistent pain, and disturbances in sleep patterns (3). This condition may cause severe disruptions in both social and occupational functioning, increase the likelihood of suicide, deteriorate general health, and lead to substantial medical costs. Consequently, it results in a marked reduction in individuals' overall quality of life (4).

Coronary heart disease is an ischemic heart condition characterized by the accumulation of atherosclerotic plaques within the coronary arteries, leading to their narrowing or obstruction (5). It is one of the primary causes of morbidity and mortality worldwide, contributing significantly to global economic strain and rising healthcare costs (6). In the United States, approximately 25% of deaths each year are attributed to coronary heart disease (7).

The co-occurrence of depression and coronary heart disease is becoming more prevalent, with each condition exacerbating the other, thus forming a detrimental cycle (8–10). Depression, as an emotional disorder, increases the risk of developing coronary heart disease and have a significant impact on their prognosis (11). The mechanisms driving this association involve several factors, including poor adherence to treatment, stimulation of the sympathetic nervous system, endothelial dysfunction, decreased heart rate variability, inflammation, and irregularities in platelet function (12). Therefore, identifying risk factors for coronary heart disease in patients with depression at an early stage and implementing targeted interventions is essential. It can reduce the likelihood of coronary heart disease in depressed individuals and improve the prognosis for those affected by both conditions.

Machine learning, as an emerging artificial intelligence tool, is essential for enhancing the accuracy of clinical disease predictions and is widely applied in the analysis of medical data (13–16). Recent studies on predictive models developed with these machine learning algorithms suggest that they demonstrate better predictive accuracy than conventional statistical approaches (17–19). Considering the complex link between depression and cardiovascular conditions like coronary heart disease, early and precise identification of coronary heart disease risk in depressed patients is crucial for reducing related adverse health effects. Regrettably, there are currently no predictive models available to evaluate the risk of coronary heart disease in individuals with depression. To address this gap, this study employs data from the National Health and Nutrition Examination Survey conducted between 2007 and 2018 to create a predictive model for assessing coronary heart disease risk in depressed patients through the use of machine learning algorithms. Personalized preventive strategy recommendations are proposed to assist clinicians in making informed clinical decisions.

## Materials and methods

### Study design and population

**Figure 1:**
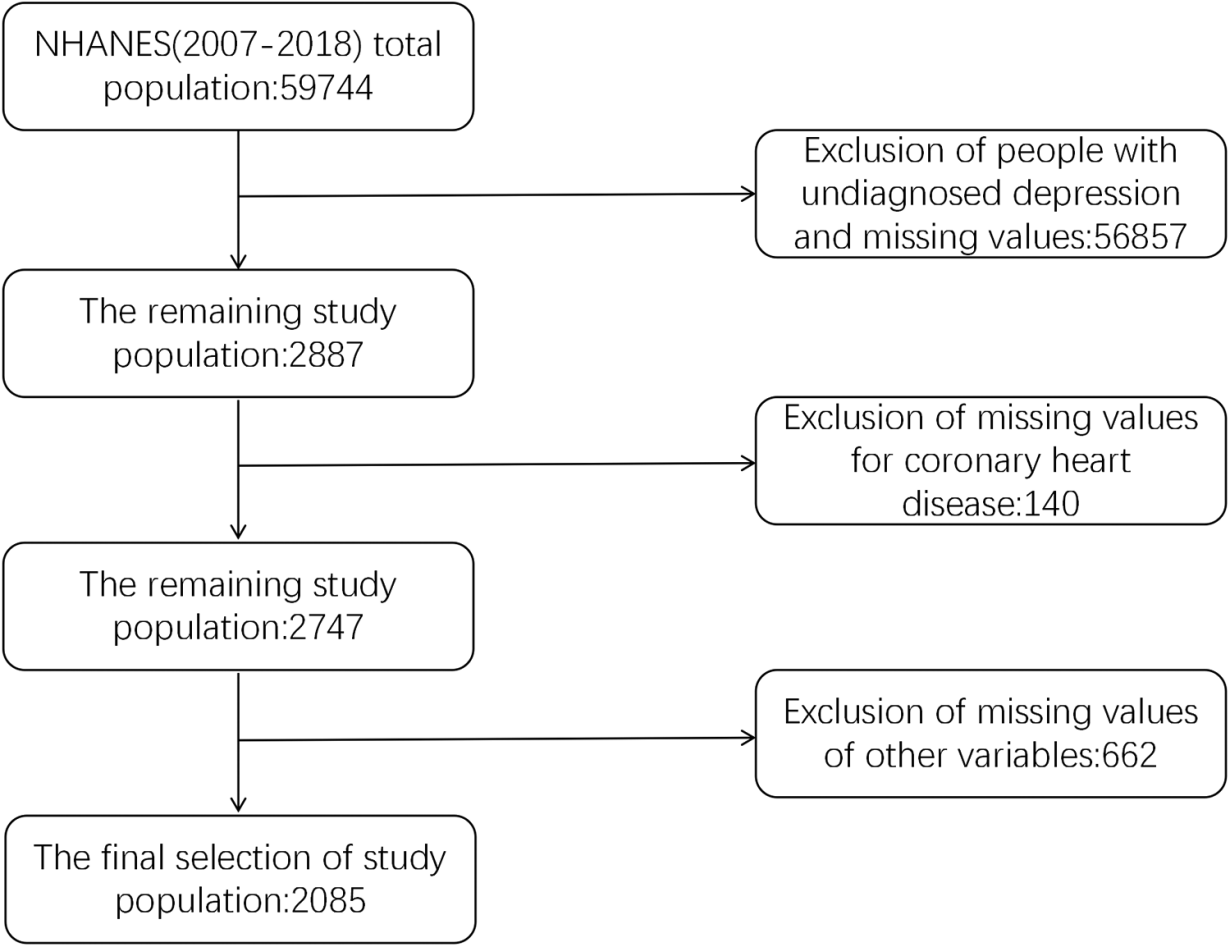
Study population screening flowchart.

The NHANES collects comprehensive background information through household screenings, interviews, and physical examinations. It provides data on the general health and nutrition of the American population, employing advanced multi-stage probability sampling methods. For this analysis, data from NHANES cycles covering the years 2007–2018 were utilized. The inclusion criteria consisted of: (1) participants with a previous diagnosis of depression; (2) individuals aged 20 years and older; and (3) complete information on all relevant variables. The exclusion criteria included: (1) participants without a previous diagnosis of depression; (2) individuals younger than 20 years; and (3) cases with missing values for any variable. Initially, 59,744 participants contributed data for the survey. Following the application of the inclusion and exclusion criteria, a final cohort of 2,085 individuals aged 20 years and older was selected for our study. The study protocol for NHANES was approved by the Institutional Review Board at the Centers for Disease Control and Prevention, with informed consent obtained from all participants. Figure 1 illustrates the screening process for the subject population.

### Study variables

This study utilized the PHQ-9 scale, a nine-question tool designed to evaluate depression. Responses are rated on a four-point Likert scale, ranging from 0 (not at all) to 3 (nearly every day), resulting in a total score that can vary from 0–27. Generally, a score of 10 or above indicates a likelihood of depression.

In the NHANES survey, participants were inquired whether a doctor or other healthcare provider had ever informed them of a diagnosis of coronary heart disease. Those who responded affirmatively were categorized as having coronary heart disease. The covariates included demographic information [age, gender, race, marital status, education level, and poverty-income ratio (PIR)], lifestyle factors (alcohol use, smoking habits, sedentary time, and sleep duration on workdays), chronic health conditions [hypertension (No/Yes), myocardial infarction (No/Yes), diabetes, chest pain (No/Yes)], “Yes” represents participants with the corresponding disease and “No” represents participants without the disease. screening data [body mass index [BMI], waist circumference [WC]], and laboratory measurements (uric acid [UA], total cholesterol [TCHOL], creatinine [CR], albumin [ALB], blood urea nitrogen [BUN], high-density lipoprotein [HDL], HbA1c, triglycerides [TG], alanine aminotransferase [ALT], and aspartate aminotransferase [AST]). Gender was categorized as male or female, race was divided into Mexican American, non-Hispanic white people, non-Hispanic black people, Hispanic people, and other races. Marital status was classified as unmarried, married or cohabitating, and married but living alone (separated, divorced, or widowed). Education levels were grouped into below 9th grade, 9th–11th grade, high school graduate, some college, or associate degree and above.

Household or individual income was modified according to the survey year and the poverty threshold specific to each state in order to determine the poverty-to-income ratio. Participants provided information on their alcohol use and smoking habits. Smoking was categorized into three distinct groups: nonsmokers, former smokers, and current smokers. Alcohol consumption was divided into five classifications: never drinkers, former drinkers, heavy drinkers (three or more drinks per day for women and four or more for men), moderate drinkers (up to two drinks per day for women and three for men), and light drinkers (not included in the other categories). Sleep duration (in hours) and sedentary time (in minutes) were assessed through a questionnaire. Medical professionals measured waist circumference and body mass index at mobile examination centers. Additional questionnaires collected data on participants' chronic conditions, including hypertension, diabetes mellitus, myocardial infarction, and chest pain. Laboratory tests provided values for UA, TCHOL, ALB, CR, HDL, BUN, TG, HbA1c, ALT, and AST.

### Development and validation of machine learning models

In this study, the dataset was randomly split into a training set and a validation set in an 8:2 ratio. Univariate and multivariate logistic regression analyses were employed to identify predictor variables. Using the training data, eight machine learning models were developed: LR, RF, GBM, SVM, XGBoost, CART, KNN, and NNET. The validation set was employed to evaluate the predictive accuracy of the models. Discrimination between models was assessed using ROC curves and PR curves, while calibration was determined through calibration curves and the Brier score, comparing predicted outcomes to actual results. The clinical applicability of the models was analyzed via DCA. To mitigate overfitting, internal validation was conducted using the Bootstrap technique. Additionally, a nomogram and web calculator derived from logistic regression was developed to visually illustrate the predictive model. Lastly, the significance of variables in the top-performing model was ranked utilizing SHAP (Shapley Additive Explanations) plots.

### Statistical analysis

Given the complex sampling design of NHANES, data were weighted during the analysis of baseline information and the logistic regression. Continuous variables were reported as means with standard errors, whereas categorical variables were presented in terms of counts and percentages. *T*-tests were utilized to assess continuous variables between the two groups, while chi-square tests or Fisher's exact tests were employed for comparing categorical variables. All statistical analyses were conducted using R software (version 4.4.1), with statistical significance defined as *P* < 0.05 for all analyses.

## Results

### Baseline characteristics

This study included 2,085 individuals with a prior diagnosis of depression, who had an average age of 46.12 years. Among these participants, 36.69% were male, 63.31% were female, 44.32% identified as non-Hispanic White persons, 20.29% as non-Hispanic Black persons, 14.58% as Mexican American, 13.29% as Hispanic American, and 7.53% as other races. Among the 2,085 participants, 124 were diagnosed with coronary heart disease, while 1,961 had no prior diagnosis of coronary heart disease.

In the training set, participants with depression were divided into two groups according to whether they had coronary heart disease. Significant statistical differences were identified between the two groups in terms of waist circumference, age, marital status, sedentary behavior, history of myocardial infarction, chest pain, hypertension, diabetes, alcohol consumption, CR, UA, ALB, BUN, TG and HbA1c (*P* < 0.05). In the validation set, significant statistical differences were found between the two groups with respect to age, sedentary time, BUN, ALT levels, marital status, history of myocardial infarction, chest pain, and diabetes (*P* < 0.05). The results are shown in Table 1.

**Table 1:** Weighted baseline characteristics of the study population.

Variables	Total	Training set		*P*-value	Validation set		*P*-value
		No	Yes		No	Yes	
N	2,085	1,569	100		392	24	
Age (years)	46.12 (0.45)	45.36 (0.50)	62.03 (1.43)	<0.001	44.44 (0.93)	63.85 (2.55)	<0.001
BMI (kg/m^2^)	30.59 (0.23)	30.28 (0.24)	31.90 (0.99)	0.11	31.53 (0.53)	31.26 (2.62)	0.92
WC (cm)	102.55 (0.56)	101.67 (0.64)	110.11 (2.35)	<0.001	104.09 (1.05)	109.07 (5.79)	0.39
PIR	2.15 (0.07)	2.13 (0.07)	1.87 (0.17)	0.13	2.27 (0.12)	2.40 (0.52)	0.8
Sleep (hours)	6.74 (0.06)	6.77 (0.07)	6.56 (0.23)	0.41	6.65 (0.15)	7.32 (0.37)	0.08
Sedentary (minutes)	389.55 (7.65)	382.83 (8.27)	461.21 (33.31)	0.02	391.39 (12.65)	547.83 (77.33)	0.04
CR (mg/dl)	0.86 (0.01)	0.85 (0.01)	0.98 (0.05)	0.01	0.87 (0.02)	0.95 (0.06)	0.24
UA (mg/dl)	5.34 (0.04)	5.31 (0.05)	5.91 (0.17)	0.001	5.33 (0.09)	5.87 (0.38)	0.16
ALB (mg/dl)	4.20 (0.01)	4.21 (0.01)	4.10 (0.05)	0.04	4.20 (0.03)	4.12 (0.06)	0.2
BUN (mg/dl)	12.70 (0.14)	12.45 (0.17)	15.68 (0.84)	<0.001	12.86 (0.30)	15.27 (1.08)	0.03
TG (mg/dl)	166.13 (3.00)	161.06 (3.22)	222.53 (27.89)	0.03	171.41 (7.73)	210.16 (31.95)	0.24
ALT (mg/dl)	26.14 (0.72)	25.14 (0.53)	36.59 (13.26)	0.39	28.23 (1.63)	19.43 (1.83)	<0.001
AST (mg/dl)	26.00 (0.62)	25.69 (0.72)	31.21 (5.88)	0.35	26.28 (0.97)	22.66 (1.68)	0.06
TCHOL (mg/dl)	195.88 (1.40)	196.20 (1.62)	190.03 (8.78)	0.49	195.39 (3.12)	205.12 (8.51)	0.28
HDL (mg/dl)	51.49 (0.46)	51.85 (0.54)	50.87 (3.95)	0.81	50.19 (0.94)	50.12 (4.33)	0.99
HbA1c	5.77 (0.03)	5.71 (0.03)	6.51 (0.18)	<0.001	5.77 (0.06)	6.81 (0.58)	0.08
Education				0.08			0.54
Less than 9th grade	275 (13.19)	178 (7.12)	17 (7.56)		67 (10.91)	3 (9.85)	
9–11th grade	431 (20.67)	290 (15.89)	27 (27.76)		83 (15.94)	9 (26.40)	
High school graduate	491 (23.55)	338 (25.90)	16 (19.63)		79 (23.57)	4 (20.45)	
Some college graduate	649 (31.13)	452 (34.33)	27 (39.30)		122 (33.56)	5 (38.78)	
College graduate or above	239 (11.46)	173 (16.76)	5 (5.74)		41 (16.03)	3 (4.52)	
Martial				<0.001			0.01
Never married	448 (21.49)	317 (22.96)	7 (5.72)		98 (23.21)	1 (2.21)	
Living with Partner	936 (44.89)	621 (47.41)	45 (50.31)		182 (51.16)	9 (42.70)	
Widowed/Divorced	701 (33.62)	493 (29.63)	40 (43.97)		112 (25.63)	14 (55.09)	
Race				0.18			0.65
Non-Hispanic White people	924 (44.32)	634 (65.62)	52 (75.49)		159 (58.17)	13 (72.28)	
Non-Hispanic Black people	423 (20.29)	298 (12.36)	14 (9.04)		92 (15.13)	4 (10.14)	
Mexican American	304 (14.58)	201 (7.45)	11 (3.59)		67 (9.19)	4 (5.78)	
Other Hispanic people	277 (13.29)	193 (7.71)	9 (4.11)		46 (7.09)	1 (2.36)	
Other race	157 (7.53)	105 (6.87)	6 (7.78)		28 (10.41)	2 (9.43)	
Sex				0.23			0.25
Female	1,320 (63.31)	919 (65.01)	48 (55.83)		243 (65.00)	9 (51.03)	
Male	765 (36.69)	512 (34.99)	44 (44.17)		149 (35.00)	15 (48.97)	
Smoke				0.43			0.53
Never	828 (39.71)	580 (38.95)	26 (31.48)		154 (38.89)	5 (38.26)	
Former	470 (22.54)	293 (20.95)	31 (28.65)		91 (23.97)	8 (13.98)	
Now	787 (37.75)	558 (40.10)	35 (39.87)		147 (37.14)	11 (47.76)	
Alcohol				0.001			0.35
Never	240 (11.51)	163 (8.27)	11 (9.59)		48 (7.95)	1 (9.38)	
Former	443 (21.25)	298 (18.35)	36 (33.93)		81 (20.47)	9 (25.08)	
Mild	530 (25.42)	359 (27.84)	29 (39.86)		98 (26.82)	9 (44.13)	
Moderate	332 (15.92)	223 (17.45)	9 (6.39)		64 (17.33)	3 (15.08)	
Heavy	540 (25.9)	388 (28.09)	7 (10.23)		101 (27.42)	2 (6.33)	
Diabetes				<0.001			0.03
No	1,392 (66.76)	989 (74.70)	30 (28.17)		253 (69.82)	11 (36.77)	
Prediabetes	168 (8.06)	118 (8.92)	7 (8.26)		32 (9.43)	3 (13.95)	
Yes	525 (25.18)	324 (16.37)	55 (63.57)		107 (20.75)	10 (49.29)	
Hypertension				0.002			0.59
No	888 (42.59)	616 (46.62)	13 (21.30)		166 (42.84)	5 (50.62)	
Yes	1,197 (57.41)	815 (53.38)	79 (78.70)		226 (57.16)	19 (49.38)	
MI				< 0.001			<0.001
No	1,941 (93.09)	1,374 (97.05)	44 (50.76)		370 (94.72)	12 (50.52)	
Yes	144 (6.91)	57 (2.95)	48 (49.24)		22 (5.28)	12 (49.48)	
CP				< 0.001			<0.001
No	1,976 (94.77)	1,399 (98.07)	53 (60.18)		377 (95.73)	15 (53.73)	
Yes	109 (5.23)	32 (1.93)	39 (39.82)		15 (4.27)	9 (46.27)	

UA, uric acid; PIR, poverty-to-income ratio; WC, waist circumference; BMI, body mass index; HDL, high density lipoprotein; TG, triglyceride; BUN, blood urea nitrogen; ALB, albumin; CR, creatinine; TCHOL, Total cholesterol; MI, myocardial infarction; CP, chest pain.

### Univariate and multivariate logistic regression analysis

To identify independent risk factors for coronary heart disease in individuals with depression, subsequent univariate and multivariate logistic regression analyses were conducted. The univariate logistic regression analysis revealed that factors such as WC, age, sedentary behavior, race, marital status, educational level, alcohol consumption, CR, UA, ALB, BUN, TG, ALT, HbA1c, diabetes, myocardial infarction, chest pain, and hypertension were significantly linked to the risk of coronary heart disease. Following this, multivariate logistic regression analysis indicated that age, education level, TG, history of myocardial infarction, and presence of chest pain emerged as independent predictive factors for coronary heart disease risk in individuals with depression (*P* < 0.05). The findings are summarized in Table 2.

**Table 2:** Weighted univariate and multivariate logistic regression analysis.

Variables	Univariate	*P*-value	Multivariate	*P*-value
	OR (95% CI)		OR (95% CI)	
Age (years)	1.08 (1.07,1.10)	<0.001	1.07 (1.05,1.10)	<0.001
BMI (kg/m²)	1.02 (0.99,1.05)	0.19	/	/
WC (cm)	1.02 (1.01,1.04)	0.001	0.99 (0.97,1.00)	0.14
PIR	0.93 (0.81,1.06)	0.28	/	/
Sleep (hours)	0.99 (0.88,1.11)	0.89	/	/
Sedentary (minute)	1.00 (1.00,1.00)	0.002	1.00 (1.00,1.00)	0.09
CR (mg/dl)	1.44 (1.13,1.84)	0.003	0.95 (0.53,1.69)	0.85
UA (mg/dl)	1.31 (1.14,1.50)	<0.001	1.15 (0.96,1.39)	0.12
ALB (mg/dl)	0.45 (0.23,0.87)	0.02	0.82 (0.36,1.86)	0.62
BUN (mg/dl)	1.07 (1.04,1.10)	<0.001	1.00 (0.95,1.05)	0.86
TG (mg/dl)	1.00 (1.00,1.00)	0.04	1.00 (1.00,1.00)	0.02
ALT (mg/dl)	1.00 (1.00,1.00)	0.03	1.00 (1.00,1.00)	0.56
AST (mg/dl)	1.00 (1.00,1.00)	0.12	/	/
TCHOL (mg/dl)	1.00 (1.00,1.00)	0.69	/	/
HDL (mg/dl)	1.00 (1.00,1.00)	0.82	/	/
HbA1c	1.43 (1.24,1.64)	<0.001	1.10 (0.90,1.34)	0.36
Education				
Less than 9th grade	Ref.	Ref.	/	/
9–11th grade	1.17 (0.60,2.26)	0.64	1.55 (0.81,2.97)	0.18
High school graduate	0.64 (0.27,1.48)	0.29	0.74 (0.26,2.10)	0.57
Some college graduate	0.73 (0.36,1.48)	0.38	1.34 (0.59, 3.07)	0.48
College graduate or above	0.22 (0.06,0.85)	0.03	0.32 (0.11, 0.91)	0.03
Martial				
Never married	Ref.	Ref.	/	/
Living with Partner	2.68 (0.94,7.68)	0.07	0.42 (0.13,1.39)	0.15
Widowed/Divorced/Separated	2.82 (1.06,7.45)	0.04	0.39 (0.12,1.27)	0.12
Race				
Non-Hispanic White people	Ref.	Ref.	/	/
Non-Hispanic Black people	0.55 (0.31,0.96)	0.04	0.66 (0.29,1.50)	0.32
Mexican American	0.42 (0.21,0.86)	0.02	0.58 (0.25,1.34)	0.20
Other Hispanic people	0.46 (0.23,0.90)	0.02	0.64 (0.22,1.82)	0.39
Other race	0.84 (0.36,1.96)	0.69	1.34 (0.54,3.30)	0.52
Sex				
Female	Ref.	Ref.	/	/
Male	1.62 (0.96,2.74)	0.07	/	/
Smoke				
Never	Ref.	Ref.	/	/
Former	1.87 (0.92,3.80)	0.08	/	/
Now	1.42 (0.73,2.77)	0.30	/	/
Alcohol				
Never	Ref.	Ref.	/	/
Former	1.90 (0.92,3.90)	0.08	0.79 (0.28,2.22)	0.65
Mild	1.68 (0.74,3.80)	0.21	1.52 (0.59,3.87)	0.38
Moderate	0.40 (0.19,0.85)	0.02	0.52 (0.17,1.56)	0.24
Heavy	0.46 (0.14,1.54)	0.21	0.49 (0.11,2.23)	0.35
MI				
No	Ref.	Ref.	Ref.	Ref.
Yes	31.79 (17.91,56.41)	<0.001	10.20 (4.60,22.60)	<0.001
Hypertension				
No	Ref.	Ref.	/	/
Yes	2.75 (1.46,5.15)	0.002	0.74 (0.33,1.71)	0.48
CP				
No	Ref.	Ref.	Ref.	Ref.
Yes	2.71 (1.65,4.44)	<0.001	11.12 (4.31,28.71)	<0.001
Diabetes				
No	Ref	Ref	/	/
Diabetes	1.44 (0.58, 3.61)	0.43	0.98 (0.34,2.80)	0.97
Yes	6.17 (3.47,10.95)	<0.001	1.75 (0.70,4.33)	0.22

UA, uric acid; PIR, poverty-to-income ratio; WC, waist circumference; BMI, body mass index; HDL, high density lipoprotein; TG, triglyceride; BUN, blood urea nitrogen; ALB, albumin; CR, creatinine; TCHOL, Total cholesterol; MI, myocardial infarction; CP, chest pain.

### Comparison of the performance of eight machine learning algorithms

**Figure 2:**
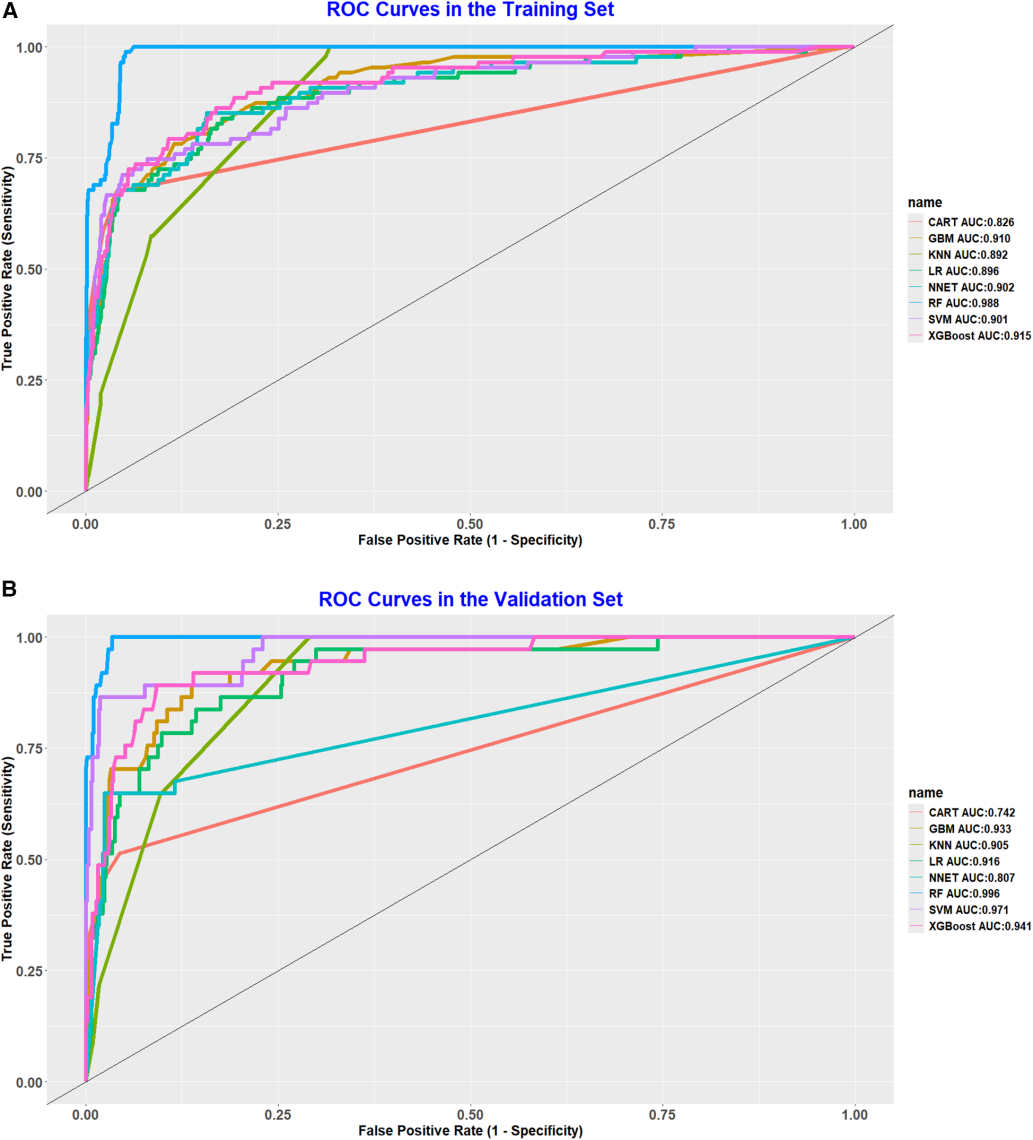
**(A)** ROC curve analysis of eight ML algorithms in the training set. **(B)** ROC curve analysis of eight ML algorithms in the validation set.

**Figure 3:**
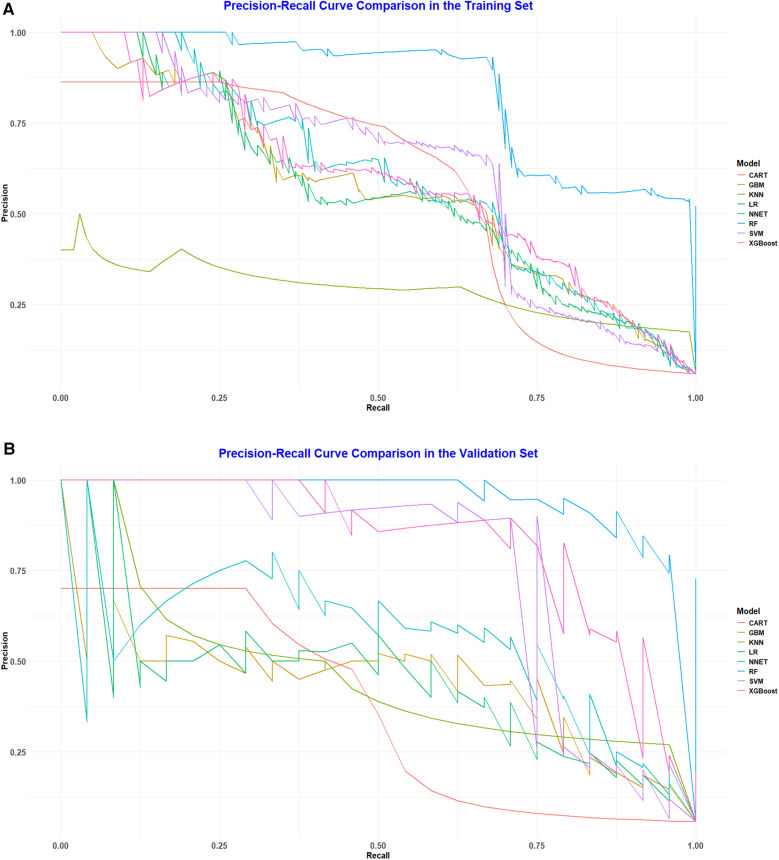
**(A)** Pr curve analysis of eight ML algorithms in the training set. **(B)** PR curve analysis of eight ML algorithms in the validation set.

To assess and validate the eight models, ROC curves were generated for all nine machine learning algorithms. In the training dataset (Figure 2A), the RF model achieved the highest AUC at 0.987, followed by the XGBoost with an AUC of 0.915, the GBM at 0.910, the NNET also at 0.902, SVM at 0.901, LR at 0.896, KNN at 0.892, and CART at 0.826. In the validation dataset (Figure 2B), the RF model maintained its leading position among the eight algorithms, with an impressive AUC of 0.996, indicating a robust discriminative capability. Additionally, in both the training set and validation set, the random forest model recorded PR curve AUC values of 0.848 and 0.960, respectively, highlighting its superior discriminative performance relative to the other models (Figure 3A,B).

**Figure 4:**
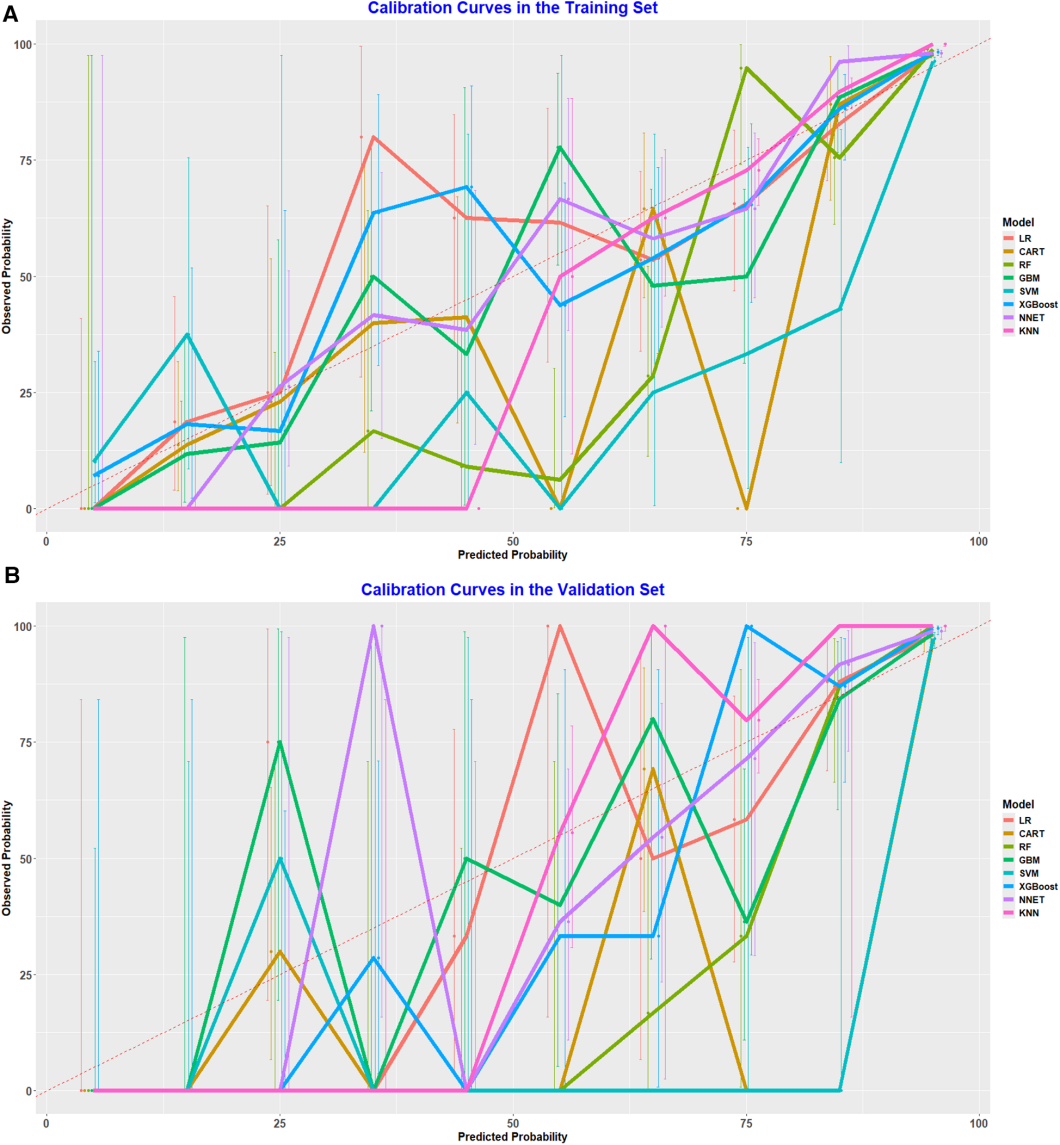
**(A)** Calibration curve analysis of eight ML algorithms in the training set. **(B)** Calibration curve analysis of eight ML algorithms in the validation.

Moreover, calibration curves from both datasets were employed to evaluate the predictive accuracy of the nine models against the actual incidence rates. The findings demonstrated a strong alignment between the actual and predicted values for the random forest model in both the training dataset (Figure 4A) and the validation dataset (Figure 4B). To further examine the model's discriminative power, Brier scores were calculated for both sets (Table 3). The random forest algorithm achieved the best Brier score of 0.026 in the training set, outperforming all other models. Likewise, in the validation set, it recorded the lowest Brier score of 0.021, further confirming its superior discriminative performance.

**Table 3:** Brier scores for training set and validation set.

Model	Brier score for training set	Brier score for validation set
KNN	0.047	0.039
CART	0.034	0.042
GBM	0.037	0.041
SVM	0.042	0.032
LR	0.038	0.041
XGBoost	0.036	0.022
NNET	0.035	0.036
RF	0.026	0.021

**Figure 5:**
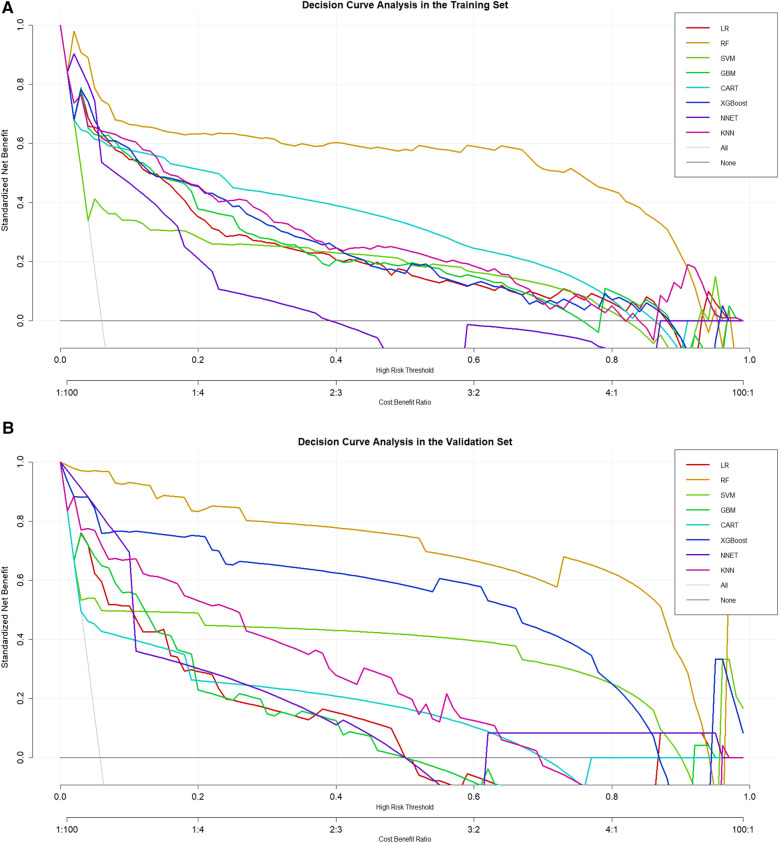
**(A)** DCA curve analysis of eight ML algorithms in the training set. **(B)** DCA curve analysis of eight ML algorithms in the validation set.

DCA was also conducted for the training set and validation set to evaluate the clinical utility of the models. The random forest model provided a significant net benefit in predicting coronary heart disease among depressed populations, further demonstrating its substantial clinical utility (Figure 5A,B).

To prevent model overfitting, the Bootstrap method was employed for internal validation, yielding an AUC of 0.864, indicating good performance. As a result, the random forest model was ultimately chosen as the predictive model for this research.

### Development of nomogram and web calculator for traditional logistic regression model

**Figure 6:**
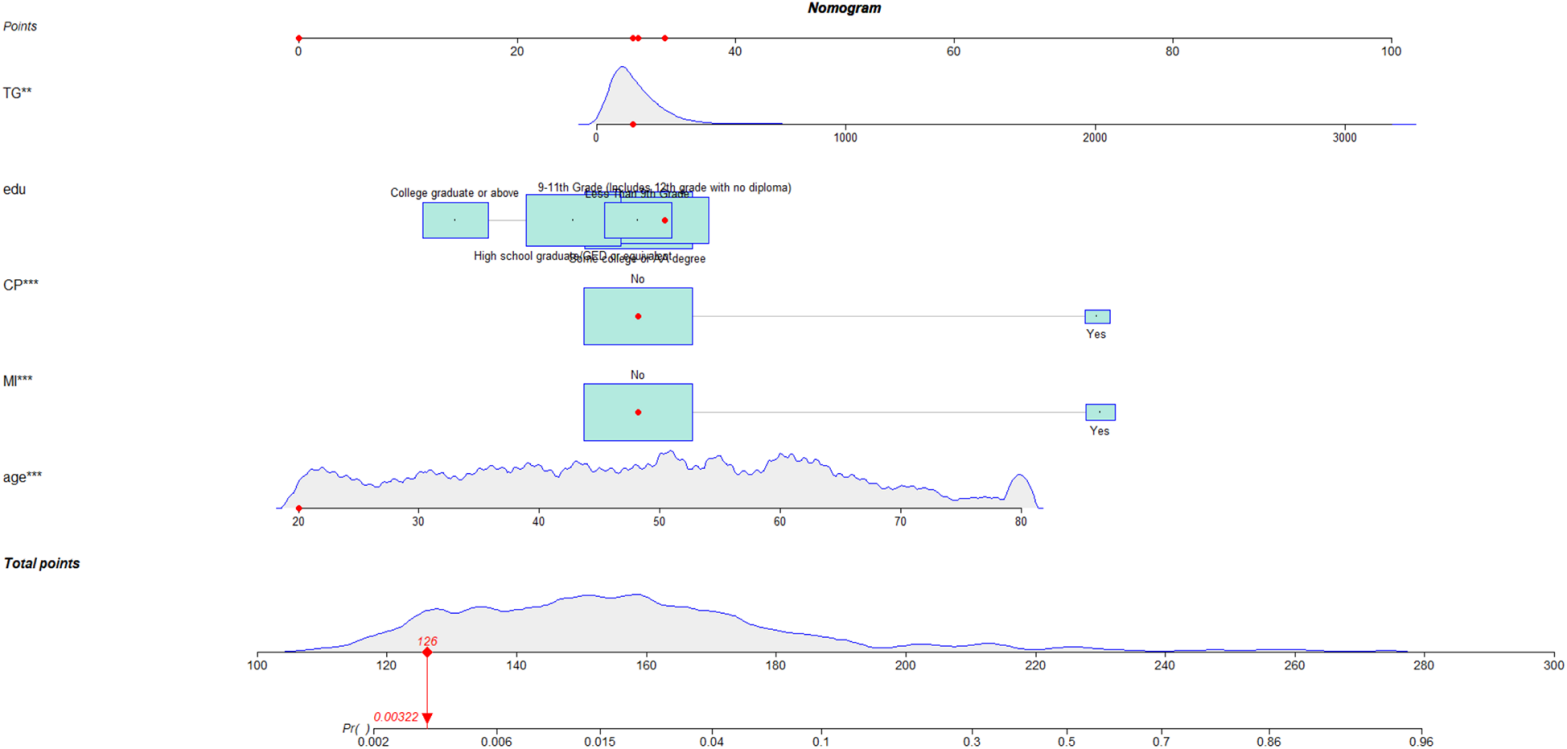
Nomogram for the risk of coronary heart disease for populations with depression.

**Figure 7:**
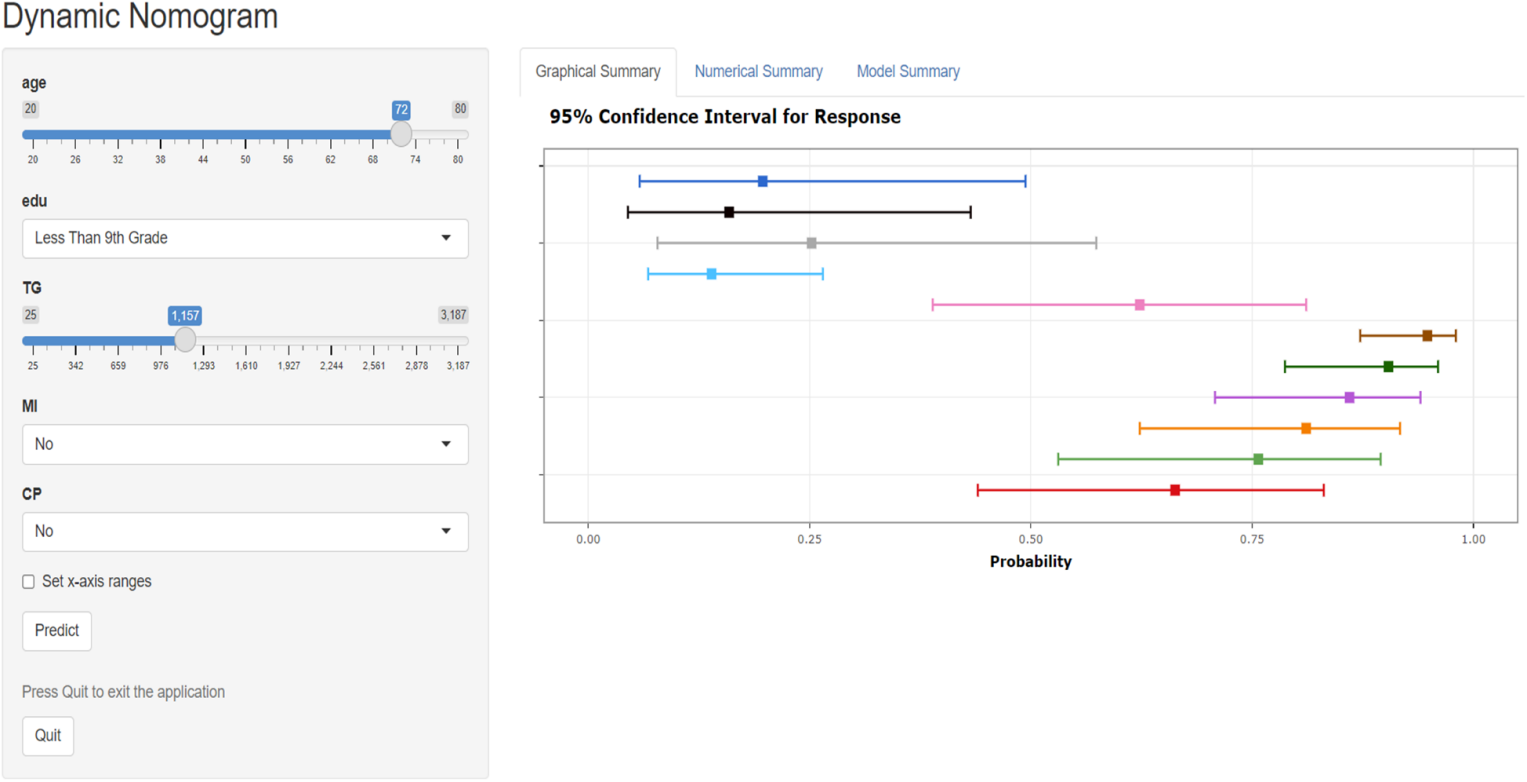
Web calculator for the risk of coronary heart disease for populations with depression.

Given the strong performance of the traditional logistic regression model in previous analyses, a nomogram was subsequently developed based on eight identified risk factors. By incorporating these eight risk factors, the nomogram enables a more precise estimation of the likelihood of specific outcomes (Figure 6). In addition, a web calculator was constructed based on the nomogram for clinicians to predict the risk of coronary heart disease in depressed patients (https://xwzxwang.shinyapps.io/DynNomapp/) (Figure 7).

### Relative importance of variables in machine learning algorithms

**Figure 8:**
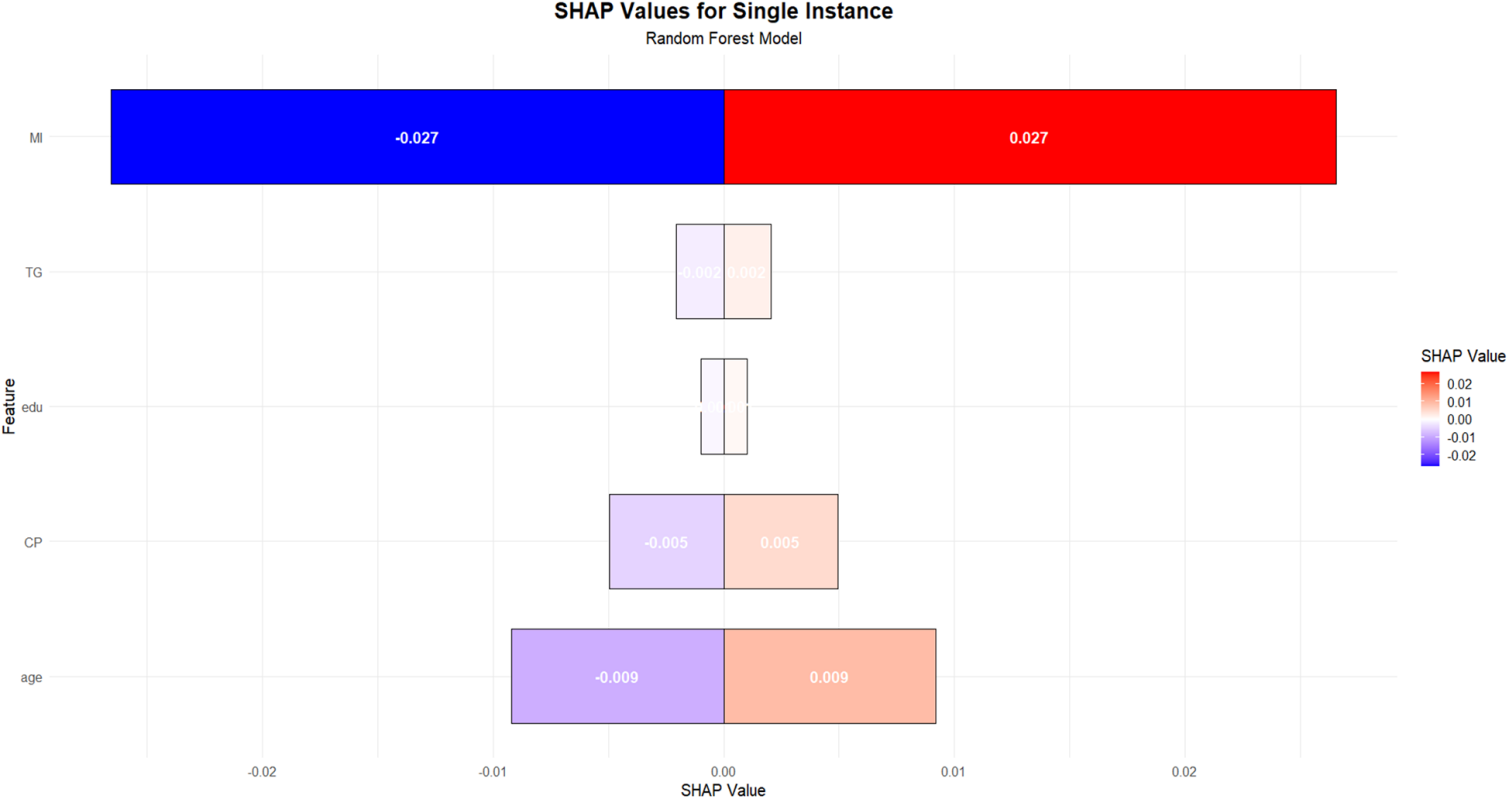
Importance ranking of variables in RF model.

Figure 8 illustrates the SHAP value interpretation for a single instance when using the random forest model to predict coronary heart disease. This figure employs a horizontal bar plot to represent the contribution of each feature to the model's prediction (SHAP value). The length and direction of each bar indicate the magnitude and direction of the feature's contribution to the prediction. Red bars represent a positive contribution towards predicting coronary heart disease, while blue bars indicate a negative contribution, suggesting a non-CHD outcome. It is evident from the figure that the feature representing MI history is the most influential, as it has the largest absolute SHAP value, showing symmetry around zero. This indicates that different values of MI introduce significant uncertainty in the model's prediction. Specifically, the SHAP value for MI is ±0.027, suggesting that variations in this feature have a substantial impact on both CHD and non-CHD predictions. The age feature follows, with a SHAP value of ±0.009, indicating a notable influence on the prediction outcome as well. In contrast, other features, such as TG, education, and chest pain, have relatively smaller SHAP values, all less than 0.005, implying their limited contribution to the model's prediction for this particular instance.

## Discussion

Our study developed and validated eight different machine learning models (LR, RF, GBM, XGB, NNET, SVM, KNN and CART) to predict the risk of coronary heart disease in individuals suffering from depression. The logistic regression was employed to identify five predictive factors: age, chest pain, myocardial infarction, education level and TCHOL. A comparative analysis was conducted focusing on the discriminative ability, calibration, and clinical applicability of each machine learning model. The findings indicated that the Random Forest model exhibited superior predictive capability compared to the other models. Clinicians can apply this machine learning-based approach to evaluate the risk of certain diseases in targeted populations.

In our research, age emerged as a significant predictor. From a physiological perspective, aging is considered an irreversible process marked by the progressive deterioration of bodily functions (20). As age progresses, the likelihood of developing coronary heart disease rises (21). It is noteworthy that this study identified a particularly significant difference between patients suffering from both depression and coronary heart disease compared to those with depression alone. The average age of patients with both conditions was more than ten years higher than that of patients with depression only. This data not only underscores the significant impact of age on disease risk but also provides valuable insights into the potential relationship between depression and coronary heart disease. Therefore, early diagnosis and treatment of chronic conditions such as coronary heart disease are crucial. Future research should further investigate how age factors influence the pathogenesis and progression of these two diseases, aiming to provide more precise and effective strategies for prevention and treatment, thereby improving the quality of life for patients.

Myocardial infarction is a significant indicator of coronary heart disease, reflecting severe pathological changes occurring within the heart and posing a critical life threat that necessitates prompt and precise medical intervention (22). The successful treatment of acute myocardial infarction hinges on prompt intervention, often requiring emergency surgery or interventional procedures to quickly restore blood flow to the coronary arteries, thereby reducing myocardial damage (23). In addition, chest pain is a common symptom of coronary heart disease. It can not only signal the existence of the disease but also act as a warning for a potential acute myocardial infarction (24). Consequently, the early identification and proper management of chest pain are essential for both preventing and treating acute myocardial infarction. There is an urgent need for further research to explore treatment options for coronary artery disease and to identify the most suitable personalized therapeutic approaches for individual patients.

Previous Mendelian randomization studies have indicated that lower educational attainment is a causal risk factor for coronary heart disease, while a genetic predisposition to higher educational attainment is associated with a reduced risk of coronary heart disease (25, 26). This finding is consistent with the results of our study, which demonstrate a significantly reduced risk of coronary heart disease in individuals with a college degree or higher education. Recent research suggests that the pathways linking educational attainment to coronary heart disease risk may involve reading comprehension skills in both genders, as well as depressive symptoms and perceived limitations, particularly in women (27). Understanding the role of education in coronary heart disease prevention is essential, and integrating educational initiatives into public health policies should be considered.

Through logistic regression analysis, our study found a positive association between triglyceride levels and the risk of developing coronary heart disease. Previous research has concluded that elevated triglyceride levels are independently associated with an increased incidence of cardiovascular events, including among patients receiving statin therapy, and hypertriglyceridemia has been established as an independent predictor of coronary heart disease risk (28, 29). This emphasizes the importance of monitoring triglyceride levels in clinical practice, as understanding the mechanisms underlying elevated triglyceride levels that lead to cardiovascular events is critical to the development of targeted treatment strategies.

The advantage of machine learning lies in its ability to train models to learn from data, offering benefits such as handling large, reliable datasets, maintaining objectivity, and ensuring reproducibility, all of which assist doctors in making more informed decisions (30–32). This study innovatively developed and validated nine machine learning algorithm models specifically designed to assess the risk of coronary heart disease in patients with depression. By evaluating various performance metrics, the RF model was selected for its superior predictive performance. Machine learning-based models can be utilized to inform clinical treatment decisions, assisting healthcare professionals in better predicting the coronary heart disease risk among depression patients and implementing necessary interventions. Furthermore, as far as we are aware, this is the first study to develop a predictive model for coronary heart disease risk in individuals with depression using machine learning methods. By employing sophisticated algorithms, this model seeks to improve early detection and intervention approaches for those experiencing both depression and cardiovascular risk factors.

There are some limitations to our study. First, since NHANES utilizes cross-sectional data, it is difficult to establish clear causal relationships between the associated diseases, as the temporal sequence of events remains unclear. Hence, Future studies that obtain longitudinal follow-up data will help to further explore the pathogenesis and disease progression of NAFLD in hypertensive patients as well as more accurately predict future risks, providing more comprehensive and in-depth guidance for clinical practice. Second, while we split the NHANES dataset into training and validation sets with a 7:3 ratio, no external datasets were used to assess the generalizability of our predictive model. Furthermore, the study population was limited to adults in the United States, which restricts the model's applicability to other global populations. Therefore, it is essential to validate the model in different countries. Third, our data were derived exclusively from the NHANES database, which relies on household interviews and health assessments conducted at Mobile Examination Centers (MEC). This dependence on a single data source could introduce bias, potentially impacting the impartiality of our findings.

## Conclusion

This study, based on the NHANES database, analyzes the independent risk factors for coronary heart disease in individuals with depression. Utilizing these risk factors, eight machine learning models—including LR, GBM, XGB, RF, NNET, SVM, KNN, and CART—were constructed and validated. After evaluating the performance of all the models, the random forest model was determined to be the best choice for prediction. The developed model can assist clinicians in identifying the risk of coronary heart disease in individuals with depression, thereby facilitating the formulation of personalized medical strategies.

## Data Availability

The raw data supporting the conclusions of this article will be made available by the authors, without undue reservation.
